# Supplementary material for: Whole exome sequencing identifies FANCM as a susceptibility gene for estrogen-receptor-negative breast cancer in Hispanic/Latina women
Source: Nat Commun. 2025 Aug 21;16:7816. doi: 10.1038/s41467-025-60564-0 (PMC12370925; doi:10.1038/s41467-025-60564-0)
Supplement: Supplementary file 1 — Supplementary Information [file 41467_2025_60564_MOESM1_ESM.pdf]

Whole exome sequencing identifies *FANCM* as a susceptibility gene for estrogen-receptor-negative breast cancer in Hispanic/Latina women

Running title:
Exome sequencing of breast cancer in Hispanic/Latina women

Jovia L. Nierenberg, PhD[1,2], Aaron W. Adamson, PhD[3], Donglei Hu, PhD[2], Scott Huntsman, MS[2], Carmina Patrick, BS[3], Min Li, BS[2], Linda Steele, MS[3], Shu Tao, PhD[4], Yuan Chun Ding, PhD[3], Barry Tong, MS[2], Yiwey Shieh, MD[5], Laura Fejerman, PhD[6,7], Stephen B. Gruber, MD[8], Christopher A. Haiman, ScD[9], Esther M. John, PhD[10,11,12], Lawrence H. Kushi, ScD[13], Gabriela Torres-Mejía, MD[14], Charité Ricker, MS[15], Jeffrey N. Weitzel, MD[16], Elad Ziv, MD[2], Susan L. Neuhausen, PhD[3]

Supplementary Materials

[1] Department of Epidemiology and Biostatistics, University of California, San Francisco, San Francisco, CA, USA.
[2] Department of Medicine, University of California, San Francisco, San Francisco, CA, USA.
[3] Department of Population Sciences, Beckman Research Institute of City of Hope, Duarte, CA, USA.
[4] Integrative Genomics Shared Resource, Beckman Research Institute of City of Hope, Duarte, CA, USA.
[5] Department of Population Health Sciences, Weill Cornell Medicine, New York, NY, USA.
[6] Department of Public Health Service, University of California, Davis, Davis, CA, USA.
[7] UC Davis Comprehensive Cancer Center, University of California, Davis, Davis, CA, USA.
[8] Department of Medical Oncology and Center for Precision Medicine, City of Hope National Medical Center, Duarte, CA, USA.
[9] Department of Preventive Medicine, Norris Comprehensive Cancer Center, Keck School of Medicine, University of Southern California, Los Angeles, CA, USA.
[10] Department of Epidemiology & Population Health, Stanford University School of Medicine, Stanford, CA, USA.
[11] Department of Medicine, Stanford University School of Medicine, Stanford, CA, USA.
[12] Stanford Cancer Institute, Stanford University School of Medicine, Stanford, CA, USA.
[13] Division of Research, Kaiser Permanente Northern California, Oakland, CA, USA.
[14] Instituto Nacional de Salud Pública, Cuernavaca, Mexico.
[15] Department of Medicine, Keck School of Medicine, University of Southern California, Los Angeles, CA, USA.
[16] Division of Precision Prevention, The University of Kansas Comprehensive Cancer Center, Kansas City, KS, USA.

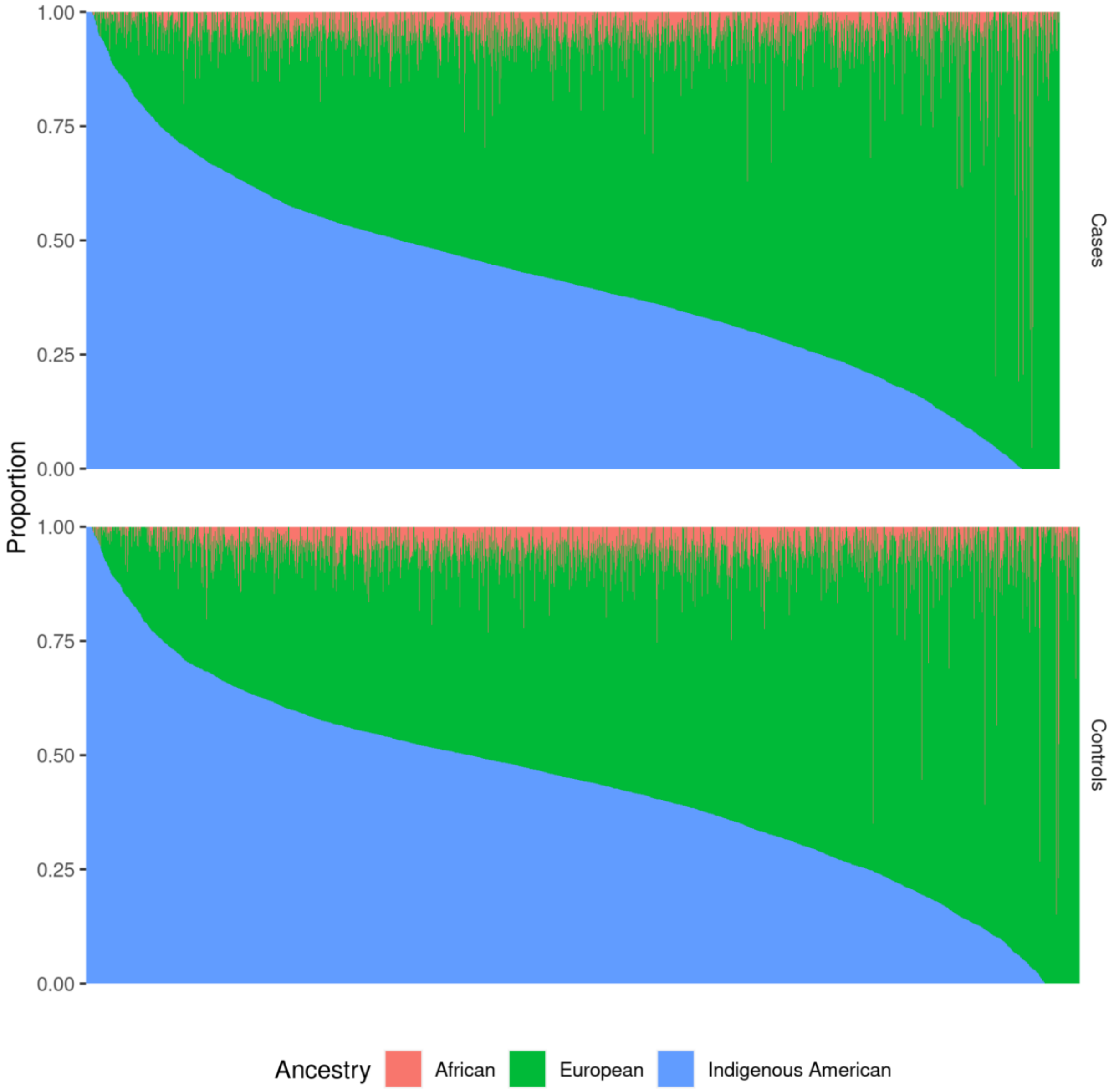

Supplementary Figure 1: Ancestry Proportions among Cases and Controls. The Y-axis shows ancestry proportion, per individual, with all ancestry proportions adding up to 1.00. Each horizontal bar represents an individual. Individuals are sorted by proportion of indigenous ancestry from most on the left to least on the right. Ancestry was calculated using ADMIXTURE 1.3. N=8,614 biologically independent samples.

A. Hereditary Studies, Overall:

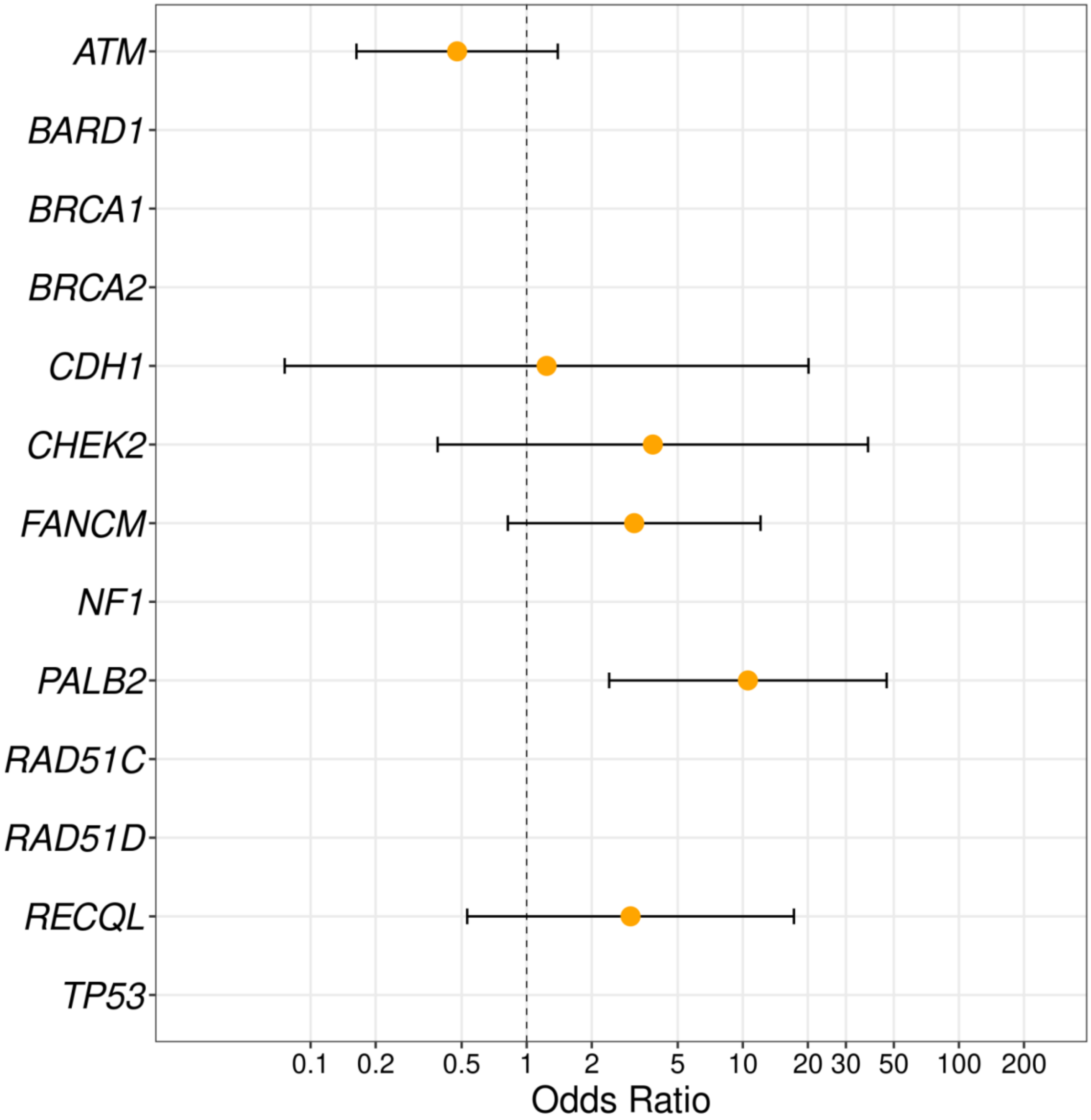

B. Hereditary Studies, Estrogen Receptor-Positive:

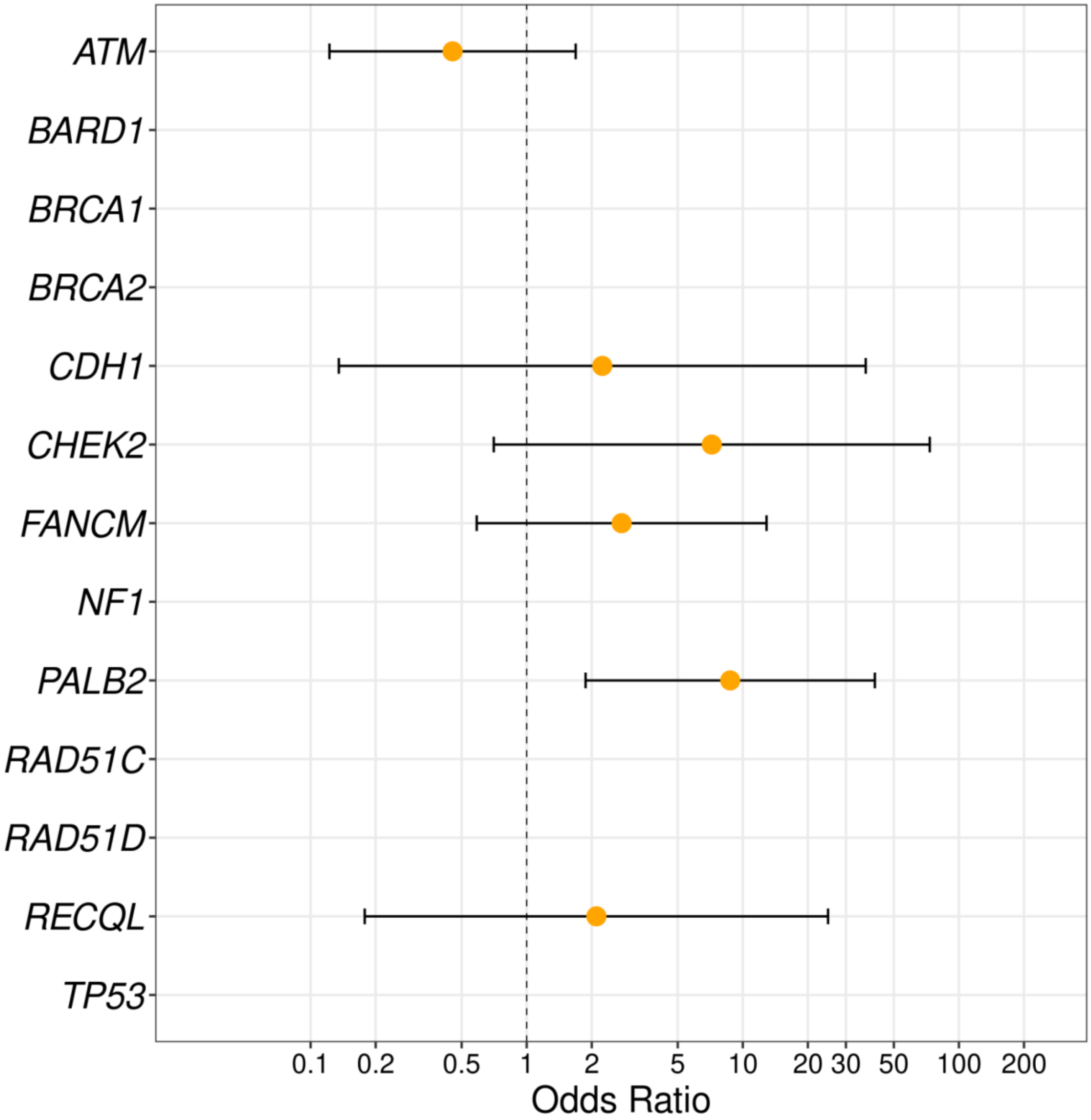

C. Hereditary Studies, Estrogen Receptor-Negative:

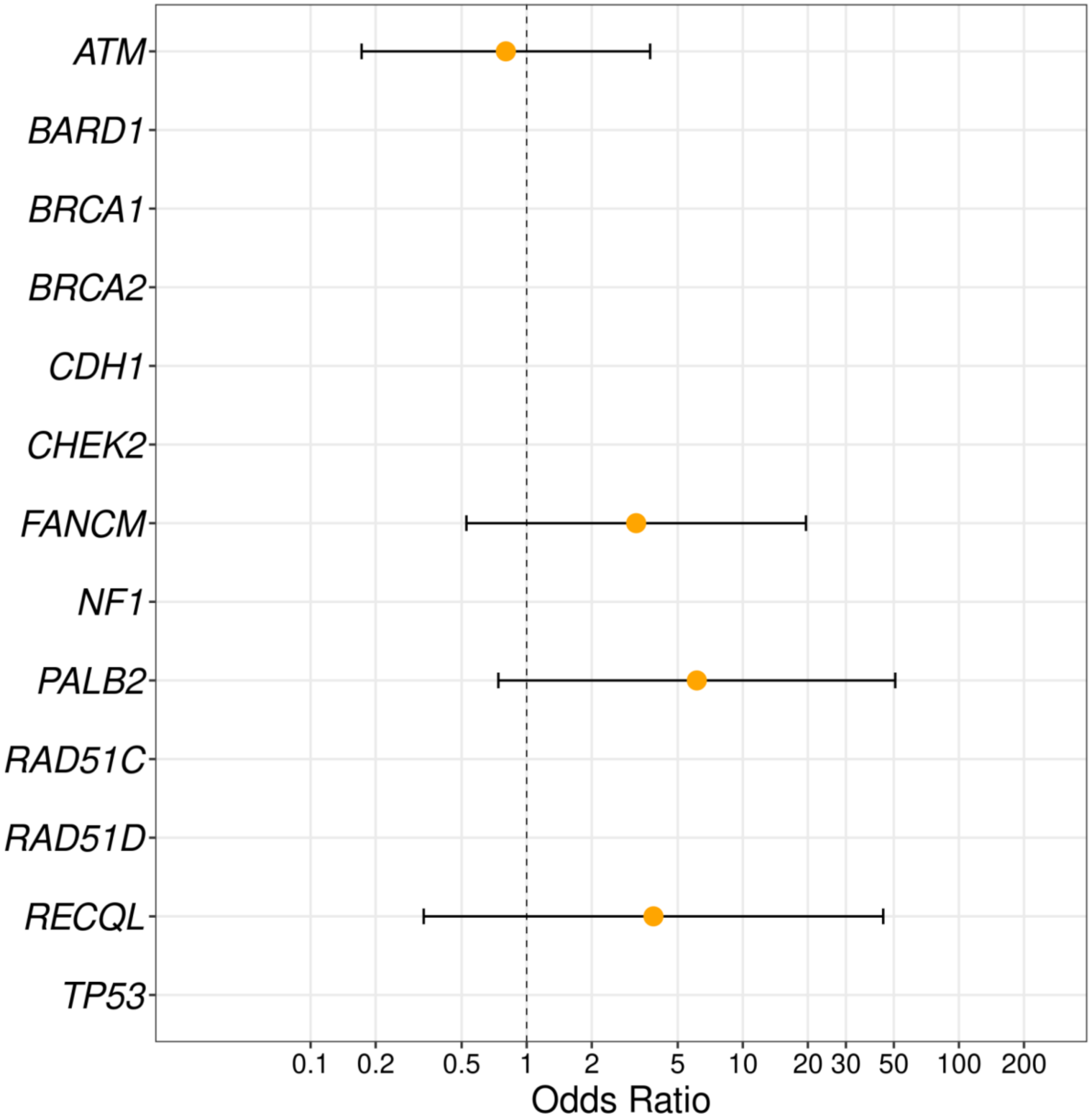

D. Unselected Studies, Overall:

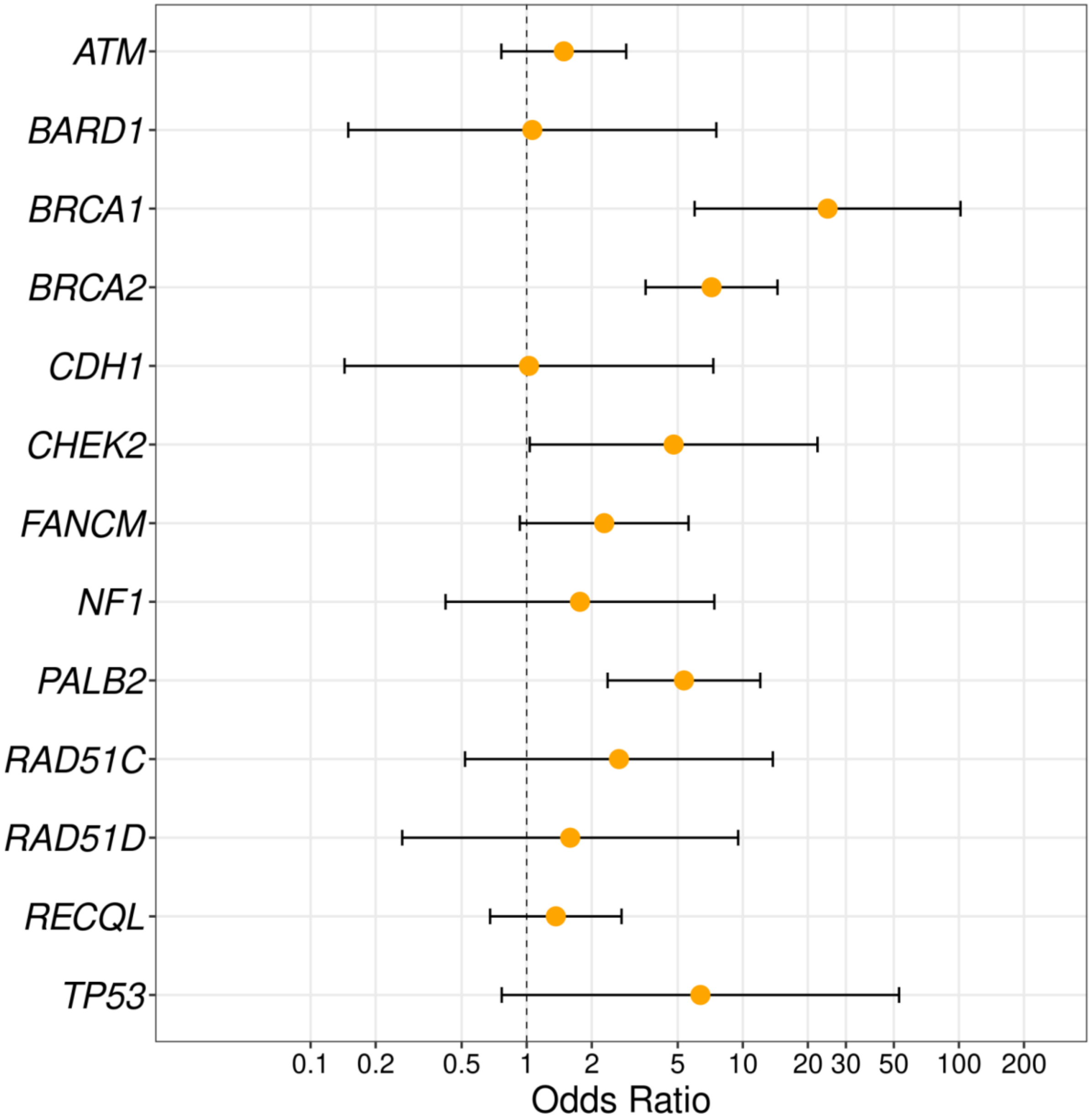

E. Unselected Studies, Estrogen Receptor-Positive:

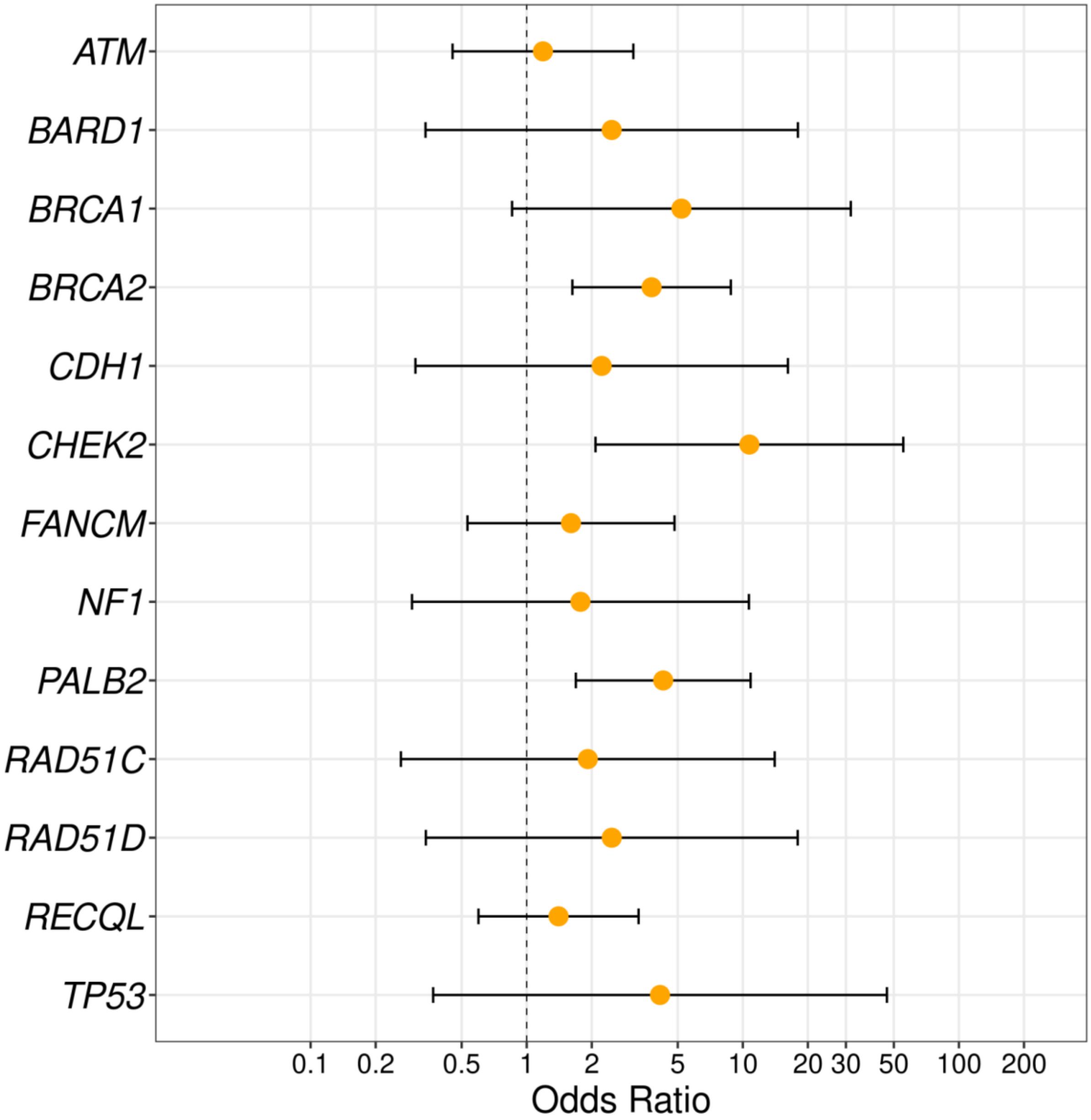

F. Unselected Studies, Estrogen Receptor-Negative:

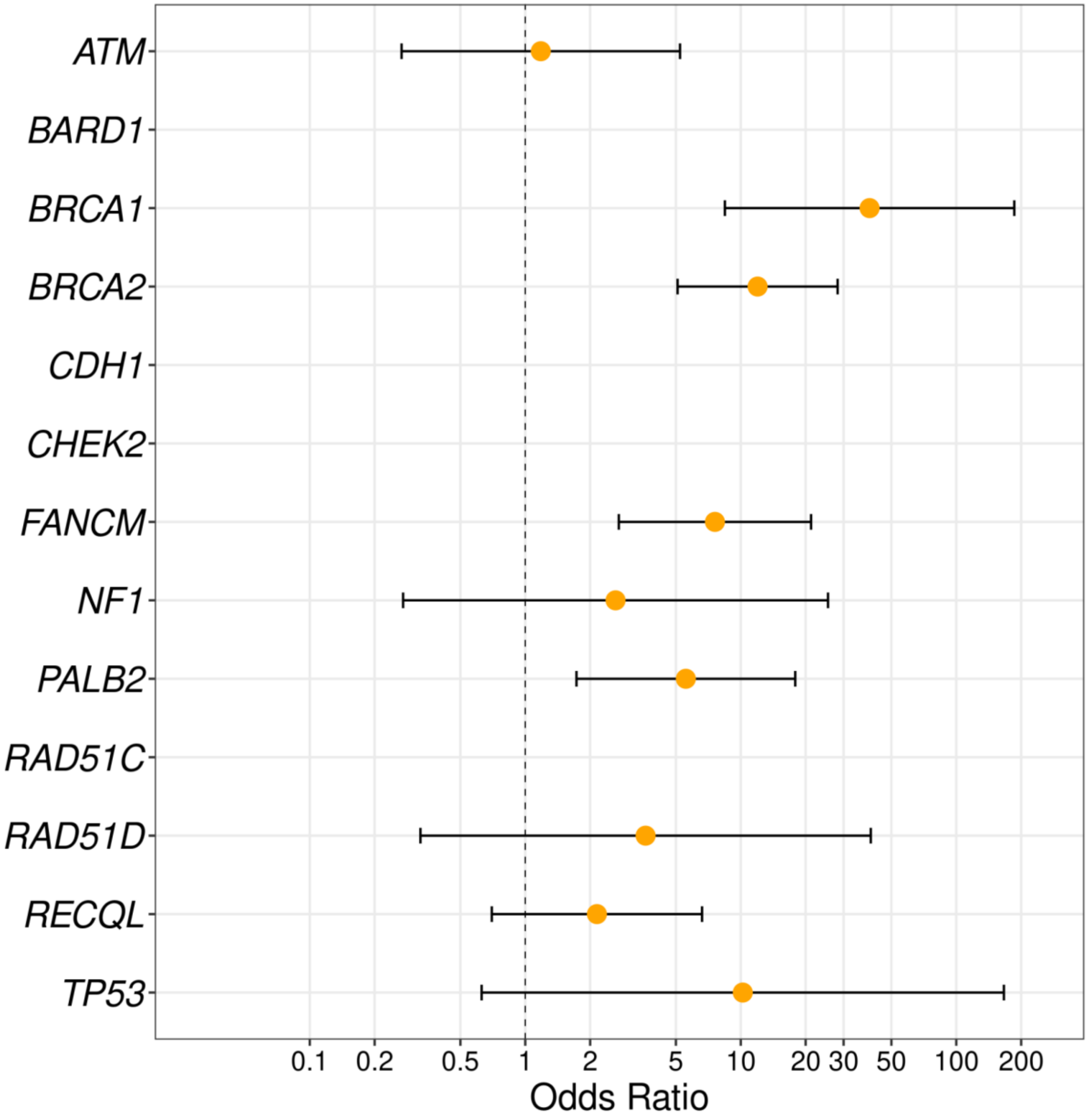

Supplementary Figure 2: Gene-Based Odds Ratios from Joint Analysis for Overall, Estrogen Receptor Positive and Estrogen Receptor Negative Disease, in Hereditary Studies and Unselected Studies Separately. Gene-based aggregate rare variant analyses were based on loss of function (LoF) variants, including frameshift, stopgain, and predicted splice variants. Odds ratios and confidence intervals from logistic regression models are presented for participants in hereditary studies with overall breast cancer (n= _ biologically independent samples, Panel A), estrogen receptor-positive (n= _ biologically independent samples, Panel B), and estrogen receptor-negative (n= _ biologically independent samples, Panel C) disease, and for participants in unselected studies with overall breast cancer (n= _ biologically independent samples, Panel D), estrogen receptor-positive (n= _ biologically

independent samples, Panel E), and estrogen receptor-negative (n= _ biologically independent samples, Panel F) disease. The orange dot represents the point estimate and the bars represent the upper and lower bounds of the 95% confidence intervals. The X-axis describes the odds ratio on a log scale, the Y-axis represents the individual genes. Genes are listed in alphabetical order. *BRCA1* and *BRCA2* are not included for hereditary studies as participants in these studies were selected for being *BRCA1/2* negative. Participants selected for hereditary risk in the Northern California Breast Cancer Family Registry were excluded from this analysis as the selection criteria were different than those used in other studies. Source data are provided as a Source Data file.

Supplementary Table 1. P-values for Gene-Based Association Testing Results for Known Genes from Joint Analysis of LoF Variants without Exome-Wide Significance, for Breast Cancer Overall, ER-positive and ER-negative Disease

Gene	Chr	All Studies		
		**Overall**	**ER-positive**	**ER-negative**
ATM	11	0.73	0.63	0.81
BARD1	2	0.44	0.17	0.67
CDH1	16	0.78	0.55	N/A
CHEK2	22	0.01	3.6×10^{-5}	0.71
NF1	17	0.62	0.46	0.37
RAD51C	17	0.39	0.34	N/A
RAD51D	17	0.27	0.27	1.9×10^{-3}
RECQL	12	0.52	0.75	0.40
TP53	17	0.11	1.00	1.6×10^{-3}

Chr=Chromosome; ER=estrogen receptor.
P-values are from gene-based SKAT-O analyses. The Bonferroni method was used to determine statistical significance, accounting for multiple comparisons. A two-sided test at an alpha threshold of $0.05/20{,}000=2.5\times10^{-6}$ was used. N=8,614 biologically independent samples.
Chr=chromosome; ER=estrogen receptor; LoF=loss of function.

Supplementary Table 2: Gene-Based P-Values from Joint Analysis of LoF Variants with Suggestive Significance, Breast Cancer Overall, ER-positive and ER-negative Disease

Gene	Chr	Overall	ER-positive	ER-negative
ACSM6	10	0.04	0.23	3.20E-03
BRCA1	17	2.30E-10	0.03	4.40E-16
BRCA2	13	8.40E-10	3.30E-04	8.00E-15
CCDC40	17	0.15	0.12	1.60E-03
CDHR2	5	0.13	0.47	3.00E-03
CEACAM8	19	0.22	0.19	4.90E-03
CHEK2	22	0.01	3.60E-05	0.71
DHRS4L2	14	3.60E-03	0.1	0.19
FANCG	9	0.15	0.29	3.50E-03
FANCM	14	9.80E-03	0.11	4.10E-07
FBP1	9	0.37	N/A	6.10E-03
FSHR	2	0.13	3.80E-03	N/A
GEMIN2	14	6.60E-03	0.07	N/A
GSTA1	6	7.90E-03	7.60E-03	0.26
LCP2	5	0.15	0.31	1.80E-03
MAPK12	22	8.30E-03	0.05	0.05
MBP	18	3.90E-04	7.60E-04	0.51
MKI67	10	0.42	0.57	3.20E-04
PALB2	16	1.80E-08	1.30E-05	5.90E-05
PRDM2	1	6.30E-03	0.02	0.57
RAD51D	17	0.27	0.27	1.90E-03
SAMD15	14	0.02	0.01	2.90E-03
SELP	1	7.10E-03	0.05	0.17
SLC26A5	7	0.06	0.02	4.40E-03
TP53	17	0.11	1	1.60E-03
TTC4	1	7.00E-03	6.00E-04	0.2
TTLL9	20	3.80E-03	4.40E-03	N/A
WDR93	15	0.03	0.56	1.60E-03
ZNF404	19	0.01	1.20E-04	N/A
ZSCAN22	19	0.03	4.60E-03	0.93

Chr=Chromosome; ER=estrogen receptor;
P-values are from gene-based SKAT-O analyses. Genes with P<0.01 in any of the three analyses are included in the table. The Bonferroni method was used to determine statistical significance, accounting for multiple comparisons. A two-sided test at an alpha threshold of $0.05/20{,}000=2.5\times10^{-6}$ was used. N=8,614 biologically independent samples.
* Discovery participants were selected for being *BRCA1/2* negative (see methods), replication results are presented for *BRCA1/2*.
Chr=chromosome; ER=estrogen receptor; LoF=loss of function.

Supplementary Table 3: Gene-Based Odds Ratios and 95% Confidence Intervals from Joint Analysis of LoF Variants for Breast Cancer Overall, ER-positive and ER-negative Disease, for Genes with Suggestive Significance

Gene	Chromosome	Overall	ER-positive	ER-negative
ACSM6	10	1.81 (0.72 - 4.54)	1.44 (0.47 - 4.39)	4.29 (1.34 - 13.74)
*BRCA1**	17	24.90 (6.05 - 102.50)	6.00 (1.08 - 33.41)	40.73 (8.90 - 186.50)
*BRCA2**	13	6.96 (3.45 - 14.03)	3.89 (1.70 - 8.89)	10.51 (4.47 - 24.73)
CCDC40	17	0.62 (0.52 - 0.74)	0.57 (0.46 - 0.70)	0.51 (0.36 - 0.73)
CDHR2	5	2.61 (0.51 - 13.46)	0.73 (0.07 - 8.06)	6.52 (0.89 - 47.68)
CEACAM8	19	2.61 (0.51 - 13.46)	2.81 (0.38 - 20.86)	7.06 (0.98 - 50.60)
CHEK2	22	4.75 (1.35 - 16.69)	9.77 (2.61 - 36.52)	NA (NA - NA)
DHRS4L2	14	1.10 (0.99 - 1.23)	1.06 (0.93 - 1.21)	1.21 (1.00 - 1.47)
FANCG	9	1.80 (0.53 - 6.16)	0.91 (0.16 - 5.10)	6.44 (1.60 - 25.98)
FANCM	14	2.68 (1.29 - 5.54)	2.20 (0.94 - 5.16)	6.69 (2.86 - 15.65)
FBP1	9	1.42 (0.24 - 8.49)	N/A	4.93 (0.69 - 35.41)
FSHR	2	5.16 (0.60 - 44.26)	12.04 (1.35 - 107.49)	N/A
GEMIN2	14	0.14 (0.02 - 1.10)	0.35 (0.04 - 2.83)	N/A
GSTA1	6	2.87 (1.28 - 6.43)	3.20 (1.32 - 7.80)	2.13 (0.56 - 8.14)
LCP2	5	5.01 (0.58 - 42.93)	4.30 (0.38 - 49.03)	12.46 (1.11 - 140.47)
MAPK12	22	0.48 (0.28 - 0.80)	0.42 (0.20 - 0.90)	0.29 (0.07 - 1.19)
MBP	18	4.05 (1.66 - 9.89)	4.31 (1.64 - 11.36)	1.80 (0.36 - 9.09)
MKI67	10	1.24 (0.53 - 2.87)	1.49 (0.56 - 3.99)	2.44 (0.76 - 7.87)
PALB2	16	6.47 (3.19 - 13.11)	5.11 (2.33 - 11.19)	6.43 (2.51 - 16.48)
PRDM2	1	1.95 (1.04 - 3.65)	2.27 (1.12 - 4.61)	1.06 (0.30 - 3.69)
RAD51D	17	2.00 (0.37 - 10.91)	2.08 (0.29 - 15.18)	5.35 (0.75 - 38.18)
SAMD15	14	1.45 (0.73 - 2.88)	1.66 (0.72 - 3.82)	3.12 (1.24 - 7.87)
SELP	1	0.23 (0.08 - 0.69)	0.23 (0.05 - 0.98)	N/A
SLC26A5	7	2.06 (1.21 - 3.52)	1.94 (1.04 - 3.59)	2.04 (0.89 - 4.67)
TP53	17	7.22 (0.89 - 58.53)	3.42 (0.30 - 38.46)	15.47 (1.39 - 172.75)
TTC4	1	2.94 (0.80 - 10.87)	4.98 (1.30 - 18.99)	1.56 (0.16 - 15.24)
TTLL9	20	0.26 (0.10 - 0.70)	0.09 (0.01 - 0.68)	N/A
WDR93	15	2.62 (0.82 - 8.35)	1.21 (0.22 - 6.68)	4.84 (1.07 - 21.91)
ZNF404	19	4.78 (1.36 - 16.80)	7.35 (1.94 - 27.90)	N/A
ZSCAN22	19	1.90 (0.95 - 3.82)	2.50 (1.18 - 5.31)	0.94 (0.21 - 4.22)

ER=estrogen receptor; LoF=loss of function.

* Discovery participants were selected for being *BRCA1/2* negative (see methods), replication results are presented for *BRCA1/2*.
N=8,614 biologically independent samples.

Supplementary Table 4: Association Between *FANCM* Variants and ER-negative Breast Cancer for Different Subsets

	Odds ratio	95% CI	P value	P value for interaction*
Age >45	10.0	3.31 - 30.39	4.3×10^{-5}	0.30
≤45	3.29	0.65 - 16.73	0.15	
<25% Indigenous American ancestry	3.67	0.81 - 16.70	0.092	0.06
25-50% Indigenous American ancestry	9.59	2.80 - 32.26	2.6×10^{-4}	
>50% Indigenous American ancestry	18.14	1.39 - 236.5	0.027	
No family history of breast cancer	5.87	1.71 - 20.09	0.00049	0.96
Family history of breast cancer	inf[&]	1.25 - inf	0.025	

* P value for interactions were derived based on an interaction term between *FANCM* variant status and either age or ancestry as a continuous variable.

[&] In analyses, comparing ER-negative breast cancer cases to controls, there were only 4 *FANCM* carriers among those with family history of breast cancer and all had ER-negative breast cancer, so the estimate of the odds ratio was derived based on exact statistics.

ER=estrogen receptor.

N=8,614 biologically independent samples including 871 cases and 1367 controls age <=45 and 2976 cases and 2791 controls age >45; 927 cases and 1048 controls Indigenous American ancestry < 25%; 1743 cases and 1845 controls with Indigenous American ancestry 25-50%; 1680 cases and 1371 controls with Indigenous American ancestry >50%; 324 cases and 891 controls with family history of breast cancer; 1939 cases and 2630 controls without family history of breast cancer.

Supplementary Table 5: Gene-Based P-Values from Joint Analysis Including LoF and Predicted Deleterious Missense Variants with Suggestive Significance for Breast Cancer Overall, ER-positive and ER-negative Disease

Gene	Chr	Overall	ER-positive	ER-negative
ACSM6	10	0.04	0.23	3.20E-03
ATR	3	0.02	9.20E-04	0.29
BRCA1	17	2.30E-10	0.03	4.40E-16
BRCA2	13	6.70E-10	2.30E-04	1.30E-14
CASP8AP2	6	2.90E-03	0.58	0.13
CCDC40	17	0.1	0.12	1.20E-04
CDHR2	5	0.13	0.47	3.00E-03
CEACAM8	19	0.22	0.19	4.90E-03
CHEK2	22	4.10E-03	1.00E-04	0.4
DDX56	7	0.09	6.00E-03	N/A
DHRS4L2	14	3.60E-03	0.1	0.19
DSTYK	1	8.70E-03	0.13	N/A
FANCG	9	0.15	0.35	8.60E-03
FANCM	14	0.04	0.2	2.90E-06
FAT3	11	0.29	0.49	6.60E-03
GEMIN2	14	7.10E-03	0.07	0.25
GSTA1	6	7.90E-03	7.60E-03	0.26
LCP2	5	0.15	0.31	1.80E-03
MBP	18	3.90E-04	7.60E-04	0.51
MKI67	10	0.42	0.57	3.20E-04
MSH6	2	4.20E-03	3.90E-03	0.04
NDOR1	9	4.70E-03	3.10E-03	0.07
PALB2	16	1.80E-08	1.30E-05	5.90E-05
PCDHGC5	5	0.21	1.0	6.50E-03
PRDM2	1	4.60E-03	0.02	0.85
PREX2	8	0.25	0.44	8.60E-03
RAD51D	17	0.44	0.17	5.40E-03
SAMD15	14	0.02	0.01	2.90E-03
SDK2	17	0.02	9.60E-03	3.00E-03
SERINC3	20	0.37	1.0	1.50E-03
SLC26A5	7	0.03	0.02	4.40E-03
TTC4	1	7.00E-03	6.00E-04	0.2
TTLL9	20	6.00E-03	4.30E-03	0.1
WDR93	15	0.03	0.56	1.60E-03
ZNF404	19	0.01	1.20E-04	N/A

Chr=Chromosome; ER=estrogen receptor; LoF=loss of function.

P-values are from gene-based SKAT-O analyses that include LoF and missense variants. The Bonferroni method was used to determine statistical significance, accounting for multiple comparisons. A two-sided test at an alpha threshold of $0.05/20,000=2.5\times10^{-6}$ was used. N=8,614 biologically independent samples.Genes with P<0.01 in any of the three analyses are included in the table. Missense variants with high likelihood of being pathogenic (VEST score > 0.8). Discovery participants were selected for being *BRCA1/2* negative (see methods), replication results are presented for *BRCA1/2*.

Supplementary Table 6: Gene-Based Odds Ratios and 95% Confidence Intervals from Joint Analysis Including Missense Variants for Breast Cancer Overall, ER-positive and ER-negative Disease, for Genes with Suggestive Significance

Gene	Chromosome	Overall	ER-positive	ER-negative
ACSM6	10	1.81 (0.72 - 4.54)	1.44 (0.47 - 4.39)	4.29 (1.34 - 13.74)
ATR	3	2.58 (1.14 - 5.87)	3.84 (1.56 - 9.45)	1.51 (0.32 - 7.20)
BRCA1	17	24.90 (6.05 - 102.50)	6.00 (1.08 - 33.41)	40.73 (8.90 - 186.50)
BRCA2	13	6.47 (3.32 - 12.63)	3.84 (1.74 - 8.46)	9.53 (4.15 - 21.85)
CASP8AP2	6	1.15 (1.05 - 1.27)	1.02 (0.90 - 1.15)	0.88 (0.73 - 1.07)
CCDC40	17	0.62 (0.53 - 0.74)	0.57 (0.46 - 0.70)	0.53 (0.37 - 0.75)
CDHR2	5	3.12 (0.63 - 15.48)	1.63 (0.23 - 11.69)	6.52 (0.89 - 47.68)
CEACAM8	19	2.61 (0.51 - 13.46)	2.81 (0.38 - 20.86)	7.06 (0.98 - 50.60)
CHEK2	22	4.01 (1.50 - 10.77)	5.98 (2.04 - 17.50)	1.31 (0.15 - 11.44)
DDX56	7	6.20 (0.75 - 51.53)	11.81 (1.34 - 104.03)	N/A
DHRS4L2	14	1.11 (1.00 - 1.23)	1.06 (0.93 - 1.21)	1.21 (1.00 - 1.47)
DSTYK	1	0.09 (0.01 - 0.70)	0.18 (0.02 - 1.37)	N/A
FANCG	9	1.43 (0.45 - 4.51)	0.72 (0.14 - 3.80)	5.01 (1.33 - 18.83)
FANCM	14	2.01 (1.08 - 3.74)	1.79 (0.85 - 3.77)	4.61 (2.13 - 9.96)
FAT3	11	1.58 (0.71 - 3.49)	1.46 (0.54 - 3.89)	3.60 (1.34 - 9.62)
GEMIN2	14	0.27 (0.06 - 1.27)	0.35 (0.04 - 2.83)	0.86 (0.11 - 6.88)
GSTA1	6	2.87 (1.28 - 6.43)	3.20 (1.32 - 7.80)	2.13 (0.56 - 8.14)
LCP2	5	5.01 (0.58 - 42.93)	4.30 (0.38 - 49.03)	12.46 (1.11 - 140.47)
MBP	18	4.05 (1.66 - 9.89)	4.31 (1.64 - 11.36)	1.80 (0.36 - 9.09)
MKI67	10	1.24 (0.53 - 2.87)	1.49 (0.56 - 3.99)	2.44 (0.76 - 7.87)
MSH6	2	0.90 (0.81 - 0.99)	1.34 (1.19 - 1.51)	1.61 (1.34 - 1.92)
NDOR1	9	3.09 (1.51 - 6.33)	3.61 (1.63 - 7.97)	3.23 (1.09 - 9.51)
PALB2	16	6.47 (3.19 - 13.11)	5.11 (2.33 - 11.19)	6.43 (2.51 - 16.48)
PCDHGC5	5	1.07 (0.98 - 1.16)	0.88 (0.79 - 0.98)	0.86 (0.73 - 1.02)
PRDM2	1	1.17 (0.71 - 1.94)	1.33 (0.73 - 2.43)	1.05 (0.40 - 2.74)
PREX2	8	1.07 (0.50 - 2.29)	0.43 (0.12 - 1.53)	2.68 (1.01 - 7.15)
RAD51D	17	1.53 (0.43 - 5.45)	2.31 (0.57 - 9.38)	2.98 (0.54 - 16.44)
SAMD15	14	1.45 (0.73 - 2.88)	1.66 (0.72 - 3.82)	3.12 (1.24 - 7.87)
SDK2	17	4.20 (0.89 - 19.78)	5.14 (0.93 - 28.36)	6.71 (0.94 - 48.04)
SERINC3	20	1.40 (0.44 - 4.41)	0.81 (0.15 - 4.34)	4.67 (1.23 - 17.76)
SLC26A5	7	2.12 (1.25 - 3.62)	1.94 (1.04 - 3.59)	2.04 (0.89 - 4.67)
TTC4	1	2.94 (0.80 - 10.87)	4.98 (1.30 - 18.99)	1.56 (0.16 - 15.24)
TTLL9	20	0.43 (0.21 - 0.91)	0.15 (0.04 - 0.64)	0.48 (0.11 - 2.03)
WDR93	15	2.84 (0.90 - 8.94)	1.65 (0.36 - 7.53)	4.84 (1.07 - 21.91)
ZNF404	19	4.78 (1.36 - 16.80)	7.35 (1.94 - 27.90)	N/A

ER=estrogen receptor
Gene-based aggregate rare variant analyses for this table were based on loss of function (LoF) variants, including frameshift, stopgain, and predicted splice variants, as well as missense variants with high likelihood of being pathogenic (VEST score > 0.8). N=8,614 biologically independent samples.
* Discovery participants were selected for being *BRCA1/2* negative (see methods), replication results are presented for *BRCA1/2*.

Supplementary Methods:

Participating Studies

Discovery cases were selected from the Clinical Cancer Genomics Community Research Network (CCGCRN),[1,2] a network of cancer centers and community-based clinics, the University of California at San Francisco (UCSF) Clinical Genetics and Prevention Program, and the University of Southern California (USC) Norris Comprehensive Cancer Center clinical genetics program. Discovery controls were self-identified H/L women enrolled by City of Hope (COH) staff through health fairs and participants in the Multiethnic Cohort (MEC), a large prospective cohort study conducted in California and Hawaii.[3] Controls from the MEC did not have BC and approximately half had diabetes.

The Cancer de Mama (CAMA) study is a population-based case–control study of BC conducted in Mexico City, Monterrey and Veracruz. Cases, aged 35–69 years at diagnosis between 2004 and 2007, were recruited from 12 hospitals (3 to 5 hospitals in each region). Controls were recruited based on membership in the same health plan as the cases and were frequency-matched on 5-year age groups.[4,5] For the California sites, we included all women who self-identified as H/L. The California Pacific Medical Center - Breast Health Center (CPMC) cohort[6] enrolled women who presented for mammography in San Francisco at CPMC between 2004 and 2011. PATHWAYS, enrolled BC cases diagnosed at Kaiser Permanente Northern California,[7]. From the nested case-control study within the MEC, we included cases with invasive BC diagnosed at the age of >50 years and controls matched on age and self-identified ethnicity.[3] The Northern California BC Family Registry (NC-BCFR)[8] recruited families and individuals with BC through the Greater Bay Area Cancer Registry. They included cases aged 18-64 with indicators of genetic susceptibility (i.e., diagnosis before age 35, history of ovarian cancer, history of BC in contralateral breast before age 50, history of BC and/or ovarian cancer in first degree relative, or history of childhood cancer cancer in first degree relative) and cases without such indicators. Controls were identified through random-digit dialing and frequency matched on race/ethnicity and 5-year age groups to cases. The San Francisco Bay Area BC Study (SFBCS),[9] a population-based multiethnic case–control study of BC, identified cases aged 35–79 years at diagnosis with invasive BC and controls as described for the NC-BCFR.

References

1. Weitzel JN, Clague J, Martir-Negron A, et al. Prevalence and Type of BRCA Mutations in Hispanics Undergoing Genetic Cancer Risk Assessment in the Southwestern United States: A Report From the Clinical Cancer Genetics Community Research Network. *J Clin Oncol*. 2013;31(2):210-216. doi:10.1200/JCO.2011.41.0027
2. MacDonald DJ, Blazer KR, Weitzel JN. Extending comprehensive cancer center expertise in clinical cancer genetics and genomics to diverse communities: the power of partnership. *J Natl Compr Cancer Netw JNCCN*. 2010;8(5):615-624. doi:10.6004/jnccn.2010.0046
3. Kolonel LN, Henderson BE, Hankin JH, et al. A multiethnic cohort in Hawaii and Los Angeles: baseline characteristics. *Am J Epidemiol*. 2000;151(4):346-357. doi:10.1093/oxfordjournals.aje.a010213
4. Angeles-Llerenas A, Ortega-Olvera C, Pérez-Rodríguez E, et al. Moderate physical activity and breast cancer risk: the effect of menopausal status. *Cancer Causes Control CCC*. 2010;21(4):577-586. doi:10.1007/s10552-009-9487-8
5. Beasley JM, Coronado GD, Livaudais J, et al. Alcohol and risk of breast cancer in Mexican women. *Cancer Causes Control CCC*. 2010;21(6):863-870. doi:10.1007/s10552-010-9513-x
6. Shieh Y, Hu D, Ma L, et al. Breast cancer risk prediction using a clinical risk model and polygenic risk score. *Breast Cancer Res Treat*. 2016;159(3):513-525. doi:10.1007/s10549-016-3953-2
7. Kwan ML, Ambrosone CB, Lee MM, et al. The Pathways Study: a prospective study of breast cancer survivorship within Kaiser Permanente Northern California. *Cancer Causes Control CCC*. 2008;19(10):1065-1076. doi:10.1007/s10552-008-9170-5
8. John EM, Sangaramoorthy M, Koo J, Whittemore AS, West DW. Enrollment and biospecimen collection in a multiethnic family cohort: the Northern California site of the Breast Cancer Family Registry. *Cancer Causes Control CCC*. 2019;30(4):395-408. doi:10.1007/s10552-019-01154-6
9. John EM, Phipps AI, Davis A, Koo J. Migration history, acculturation, and breast cancer risk in Hispanic women. *Cancer Epidemiol Biomark Prev Publ Am Assoc Cancer Res Cosponsored Am Soc Prev Oncol*. 2005;14(12):2905-2913. doi:10.1158/1055-9965.EPI-05-0483
