# Supplementary material for: Whole exome sequencing identifies FANCM as a susceptibility gene for estrogen-receptor-negative breast cancer in Hispanic/Latina women
Source: Nat Commun. 2025 Aug 21;16:7816. doi: 10.1038/s41467-025-60564-0 (PMC12370925; doi:10.1038/s41467-025-60564-0)
Supplement: Supplementary file 3 — Description of Additional Supplementary Files [file 41467_2025_60564_MOESM3_ESM.pdf]

Description of Additional Supplementary Files

Supplementary Data 1: Gene list selected for replication. This file includes only one sheet with one column which includes 857 genes which were targeted as part of the replication phase of the study.

Supplementary Data 2: Sample list and fold-coverage for discovery and replication. This file includes 2 sheets. One sheet includes the list of all participants included in the discovery phase (whole exome sequencing) and their fold coverage. The second sheet includes the list of all participants including the replication phase and their fold coverage. Data include the center contributing the samples, the case-control status (case=1, control=0), the age (defined as age at diagnosis for cases and age at recruitment for controls), family history of breast cancer (yes=1, no=0), estrogen receptor status (yes=1, no=0), Her2 status (yes=1, no=0), triple negative breast cancer (tnbc, yes=1, no=0) and fold coverage. NA for all data fields means missing data.

Supplementary Data 3: Loss of function variants in genes with exome wide significance in at least one analysis. This file contains one sheet with a list of the loss of function variant. The columns include gene name, chromosome number, base pair (in Build39/Hg19), allele A1, allele A2, the predicted region of the gene affected (exonic or splicing), the predicted effect of the variant (stopgain, frameshift insertion, frameshift deletion, exon 9-12 delection), Clinvar designation (missing = .), CADD prediction of deleteriousness, allele count in cases and allele count in controls.
