# Supplementary material for: Whole exome sequencing identifies FANCM as a susceptibility gene for estrogen-receptor-negative breast cancer in Hispanic/Latina women
Source: Nat Commun. 2025 Aug 21;16:7816. doi: 10.1038/s41467-025-60564-0 (PMC12370925; doi:10.1038/s41467-025-60564-0)
Supplement: Supplementary file 7 — Reporting summary [file 41467_2025_60564_MOESM7_ESM.pdf]

nature portfolio

Corresponding author(s): Elad Ziv

Double-anonymous peer review submissions: write DAPR and your manuscript number here instead of author names.

Last updated by author(s): *YYYY-MM-DD* 2025/5/2

Reporting Summary

Nature Portfolio wishes to improve the reproducibility of the work that we publish. This form provides structure for consistency and transparency in reporting. For further information on Nature Portfolio policies, see our [Editorial Policies] and the [Editorial Policy Checklist].

Statistics

For all statistical analyses, confirm that the following items are present in the figure legend, table legend, main text, or Methods section.

n/a	Confirmed	
☐	☒	The exact sample size (*n*) for each experimental group/condition, given as a discrete number and unit of measurement
☐	☒	A statement on whether measurements were taken from distinct samples or whether the same sample was measured repeatedly
☐	☒	The statistical test(s) used AND whether they are one- or two-sided<br>*Only common tests should be described solely by name; describe more complex techniques in the Methods section.*
☐	☒	A description of all covariates tested
☐	☒	A description of any assumptions or corrections, such as tests of normality and adjustment for multiple comparisons
☐	☒	A full description of the statistical parameters including central tendency (e.g. means) or other basic estimates (e.g. regression coefficient) AND variation (e.g. standard deviation) or associated estimates of uncertainty (e.g. confidence intervals)
☐	☒	For null hypothesis testing, the test statistic (e.g. *F*, *t*, *r*) with confidence intervals, effect sizes, degrees of freedom and *P* value noted<br>*Give P values as exact values whenever suitable.*
☒	☐	For Bayesian analysis, information on the choice of priors and Markov chain Monte Carlo settings
☒	☐	For hierarchical and complex designs, identification of the appropriate level for tests and full reporting of outcomes
☐	☒	Estimates of effect sizes (e.g. Cohen's *d*, Pearson's *r*), indicating how they were calculated

Our web collection on [statistics for biologists] contains articles on many of the points above.

Software and code

Policy information about [availability of computer code]

Data collection	Paired-end reads were aligned to human reference genome (hg37) using the Burrows-Wheeler Alignment Tool (BWA, version 0.7.5a-r405) under default settings, and the aligned binary format sequence (BAM) files were sorted and indexed using SAMtools. Duplicate reads were removed from the sorted and indexed BAMs using Picard MarkDuplicates (version 1.67, http://broadinstitute.github.io/picard/). Variant calling from BAM files from COH IGC and the Broad Sequencing Center were performed together using GATK HaplotypeCaller (https://software.broadinstitute.org/gatk) after local realignment of reads around insertions and deletions (indels) and base quality score recalibration by The Genome Analysis Toolkit (GATK, v3.6-0-g89b7209). Variants were annotated using ANNOVAR (version 2017-07-17 01:17:05 -0400 (Mon, 17 Jul 2017)). We used PLINK 1.9 (http://www.cog-genomics.org/plink/1.9/)28 to exclude first-degree relatives or duplicate samples within the discovery and replication samples.
Data analysis	We estimated genetic ancestry using ADMIXTURE 1.3. Gene-based aggregate rare variant analyses were conducted using R, version 4.2.2 (2022-10-31). Statistical significance was determined using the SKAT-O in the SKAT R package, version 2.2.4. Odds ratios (OR) and 95% confidence intervals (CI) for each gene were calculated using the glm function in R.

For manuscripts utilizing custom algorithms or software that are central to the research but not yet described in published literature, software must be made available to editors and reviewers. We strongly encourage code deposition in a community repository (e.g. GitHub). See the Nature Portfolio [guidelines for submitting code & software] for further information.

Data

Policy information about availability of data

All manuscripts must include a data availability statement. This statement should provide the following information, where applicable:

- Accession codes, unique identifiers, or web links for publicly available datasets
- A description of any restrictions on data availability
- For clinical datasets or third party data, please ensure that the statement adheres to our policy

Data will be made available to academic researchers on dbGaP. https://www.ncbi.nlm.nih.gov/projects/gap/cgi-bin/study.cgi?study_id=phs003144.v1.p1

Research involving human participants, their data, or biological material

Policy information about studies with human participants or human data. See also policy information about sex, gender (identity/presentation), and sexual orientation and race, ethnicity and racism.

Reporting on sex and gender	Our study included female participants by biological sex assessed using genetic data.
Reporting on race, ethnicity, or other socially relevant groupings	Our study included self-identified Hispanic/Latino participants.
Population characteristics	Our study included cases with a breast cancer diagnosis and controls without a breast cancer diagnosis.
Recruitment	Discovery cases were selected from the Clinical Cancer Genomics Community Research Network (CCGCRN),1,2 a network of cancer centers and community-based clinics, the University of California at San Francisco (UCSF) Clinical Genetics and Prevention Program, and the University of Southern California (USC) Norris Comprehensive Cancer Center clinical genetics program. Discovery controls were self-identified H/L women enrolled by City of Hope (COH) staff through health fairs and participants in the Multiethnic Cohort (MEC), a large prospective cohort study conducted in California and Hawaii.3 Controls from the MEC did not have BC and approximately half had diabetes.<br><br>The Cancer de Mama (CAMA) study is a population-based case–control study of BC conducted in Mexico City, Monterrey and Veracruz. Cases, aged 35–69 years at diagnosis between 2004 and 2007, were recruited from 12 hospitals (3 to 5 hospitals in each region). Controls were recruited based on membership in the same health plan as the cases and were frequency-matched on 5-year age groups.4,5 For the California sites, we included all women who self-identified as H/L. The California Pacific Medical Center - Breast Health Center (CPMC) cohort6 enrolled women who presented for mammography in San Francisco at CPMC between 2004 and 2011. PATHWAYS, enrolled BC cases diagnosed at Kaiser Permanente Northern California,7. From the nested case-control study within the MEC, we included cases with invasive BC diagnosed at the age of >50 years and controls matched on age and self-identified ethnicity.3 The Northern California BC Family Registry (NC-BCFR)8 recruited families and individuals with BC through the Greater Bay Area Cancer Registry. They included cases aged 18-64 with indicators of genetic susceptibility (i.e., diagnosis before age 35, history of ovarian cancer, history of BC in contralateral breast before age 50, history of BC and/or ovarian cancer in first degree relative, or history of childhood cancer cancer in first degree relative) and cases without such indicators. Controls were identified through random-digit dialing and frequency matched on race/ethnicity and 5-year age groups to cases. The San Francisco Bay Area BC Study (SFBCS),9 a population-based multiethnic case–control study of BC, identified cases aged 35–79 years at diagnosis with invasive BC and controls as described for the NC-BCFR. The mean age of cases is 52.1 The mean age of controls is 55.9.
Ethics oversight	All participants were consented and enrolled through center-specific institutional review board-approved protocols.

Note that full information on the approval of the study protocol must also be provided in the manuscript.

Field-specific reporting

Please select the one below that is the best fit for your research. If you are not sure, read the appropriate sections before making your selection.

☒ Life sciences ☐ Behavioural & social sciences ☐ Ecological, evolutionary & environmental sciences

For a reference copy of the document with all sections, see nature.com/documents/nr-reporting-summary-flat.pdf

Life sciences study design

All studies must disclose on these points even when the disclosure is negative.

Sample size	We used all available data.
Data exclusions	We excluded participants with <20-fold average coverage, discovery cases with previously undetected BRCA1/2 pathogenic variants, and first-degree relatives or duplicate samples within the discovery and replication samples.
Replication	Exome sequencing was performed using a discovery and replication design, however, our primary findings are from a combined analysis

Replication	(including both discovery and replication participants). Additional replication was not feasible due to lack of available data in this under-represented population.
Randomization	Not applicable, as this was a case-control study.
Blinding	Not applicable, as this was a case-control study.

Reporting for specific materials, systems and methods

We require information from authors about some types of materials, experimental systems and methods used in many studies. Here, indicate whether each material, system or method listed is relevant to your study. If you are not sure if a list item applies to your research, read the appropriate section before selecting a response.

Materials & experimental systems

n/a	Involved in the study
☒	☐ Antibodies
☒	☐ Eukaryotic cell lines
☒	☐ Palaeontology and archaeology
☒	☐ Animals and other organisms
☒	☐ Clinical data
☒	☐ Dual use research of concern
☒	☐ Plants

Methods

n/a	Involved in the study
☒	☐ ChIP-seq
☒	☐ Flow cytometry
☒	☐ MRI-based neuroimaging
